# Adverse childhood experiences, mental distress, and autoimmune disease in adult women: findings from two large cohort studies

**DOI:** 10.1017/S0033291724003544

**Published:** 2025-02-11

**Authors:** Ole Köhler-Forsberg, Fenfen Ge, Thor Aspelund, Yue Wang, Fang Fang, Gunnar Tomasson, Edda Thordadottir, Arna Hauksdóttir, Huan Song, Unnur A. Valdimarsdottir

**Affiliations:** 1 Psychosis Research Unit, Aarhus University Hospital – Psychiatry, Aarhus, Denmark; 2 Department of Clinical Medicine, Aarhus University, Aarhus, Denmark; 3 Centre of Public Health Sciences, Faculty of Medicine, University of Iceland, Reykjavík, Iceland; 4 The National Centre for Register-based Research, Aarhus University, Aarhus, Denmark; 5 Department of Medical Epidemiology & Biostatistics, Karolinska Institutet, Stockholm, Sweden; 6 West China Biomedical Big Data Center, West China Hospital, Sichuan University, Chengdu, China; 7 Harvard T.H. Chan School of Public Health, Boston, USA

**Keywords:** adverse childhood experiences, anxiety, autoimmune diseases, depression, mediation, PTSD

## Abstract

**Background:**

Adverse childhood experiences (ACEs) have been associated with increased risks of autoimmune diseases. However, data are scarce on the role of specific ACEs as well as the potential mediating role of adverse mental health symptoms in this association.

**Methods:**

A cohort study using the nationwide Icelandic Stress-And-Gene-Analysis (SAGA, 22,423 women) cohort and the UK Biobank (UKB, 86,492 women) was conducted. Participants self-reported on five ACEs. Twelve autoimmune diseases were self-reported in SAGA and identified via hospital records in UKB. Poisson regression was used to assess the cross-sectional association between ACEs and autoimmune diseases in both cohorts. Using longitudinal data on self-reported mental health symptoms in the UKB, we used causal mediation analyses to study potential mediation by depressive, anxiety, and PTSD symptoms in the association between ACEs and autoimmune diseases.

**Results:**

The prevalence of ACEs was 50% in SAGA and 35% in UKB, while the prevalence of autoimmune diseases was 29% (self-reported) and 14% (clinically confirmed), respectively. In both cohorts, ACEs were associated with an increased prevalence ratio (PR) of any studied autoimmune disease in a dose–response manner (PR = 1.10 (95%CI = 1.08–1.12) per ACE), particularly for Sjögrens (PR = 1.34), polymyalgia rheumatica (PR = 1.20), rheumatoid arthritis (PR = 1.14), systemic lupus erythematosus (PR = 1.13), and thyroid disease (PR = 1.11). Sexual abuse and physical and emotional neglect were consistently associated with an elevated prevalence of autoimmune diseases when including all ACEs in the model. Approximately one fourth of the association was mediated through depression, anxiety, and PTSD.

**Conclusions:**

These findings based on two large cohorts indicate a role of ACEs and corresponding mental health distress in autoimmune diseases among adult women.

## Background

Adverse childhood experiences (ACEs) represent severe early-life stressors that frequently occur in the general population (Felitti et al., 1998) and have been associated with an increased risk of multiple mental and somatic health outcomes (Chen et al., 2010; Danielsdottir et al., 2022; Dube et al., 2009; Felitti et al., 1998; LeMoult et al., 2020; Morris et al., 2019; Peh et al., 2019; Rubinstein et al., 2020; Yang et al., 2022). Several studies have reported a link between ACEs and mental disorders (Chen et al., 2010; Danese et al., 2009; Dragioti et al., 2022; Köhler-Forsberg et al., 2024; LeMoult et al., 2020; Peh et al., 2019), particularly with mood and anxiety disorders and with specific ACEs such as emotional and sexual abuse contributing to a significant risk increase (Dragioti et al., 2022; Köhler-Forsberg et al., 2024; LeMoult et al., 2020). Furthermore, ACEs can lead to a chronic stress-response and hence chronic increased immune system activity and inflammation (Danese et al., 2009; Rasmussen et al., 2020), which may represent a mechanistic link to the reported elevated rates of autoimmune diseases among individuals exposed to ACEs (Dube et al., 2009; Morris et al., 2019; Rubinstein et al., 2020), although other findings did not support such an association (Gatto et al., 2022). Men and women exhibit distinct patterns of ACEs, with women experiencing more complex and varied ACEs (Haahr-Pedersen et al., 2020) and showing a stronger association between ACEs and inflammatory markers compared to men (Pitts et al., 2024).

The comorbidity of autoimmune diseases and mental disorders is highly multifactorial and affected by both genetic and environmental factors (Danese et al., 2009; Felitti et al., 1998; Soares et al., 2021). Nevertheless, although genetic studies have suggested that ACEs can lead to epigenetic changes affecting both mental and physical health in adulthood (Houtepen et al., 2018; Neves et al., 2021; Parel & Peña, 2022), the associations between ACEs, mental health symptoms and autoimmune diseases have been little studied within the same setting. A recent study on 306 adults with an autoimmune disease and 292 healthy controls found that the relationship between childhood trauma and autoimmune diseases was mediated by the specific mental health symptoms dissociation and alexithymia (Macarenco et al., 2021). However, to our knowledge no large cohort studies have investigated whether the association between ACEs and autoimmune diseases potentially is mediated by mental health symptoms that are more frequently occurring in the population, such as mood and anxiety symptoms. Such potential mediation may not only represent a direct mediation by mental distress but also an underlying mediation of associated biologic sequalae, for example, chronic inflammation.

As ACEs, mood and anxiety symptoms, and autoimmune diseases frequently occur at a population level, it is important to study the potential mediating role of these mental health symptoms in the association between ACEs and autoimmune diseases. To this end, we leveraged two large cohorts from two North European countries to study the association between ACEs and the risk of autoimmune diseases among adult women, as well as potential mediation by mood and anxiety symptoms.

## Methods

We used data from two population-based cohorts: The Stress-And-Gene-Analysis (SAGA, approved by the National Bioethics Committee: 17–238) cohort on Icelandic women and the United Kingdom Biobank (UKB, approved by the Ethics Review Authority (2022-01516-01) in Sweden) on participants from the UK. The authors assert that all procedures contributing to this work comply with the ethical standards of the relevant national and institutional committees on human experimentation and with the Helsinki Declaration of 1975, as revised in 2008.

### The SAGA cohort

**Figure 1.**
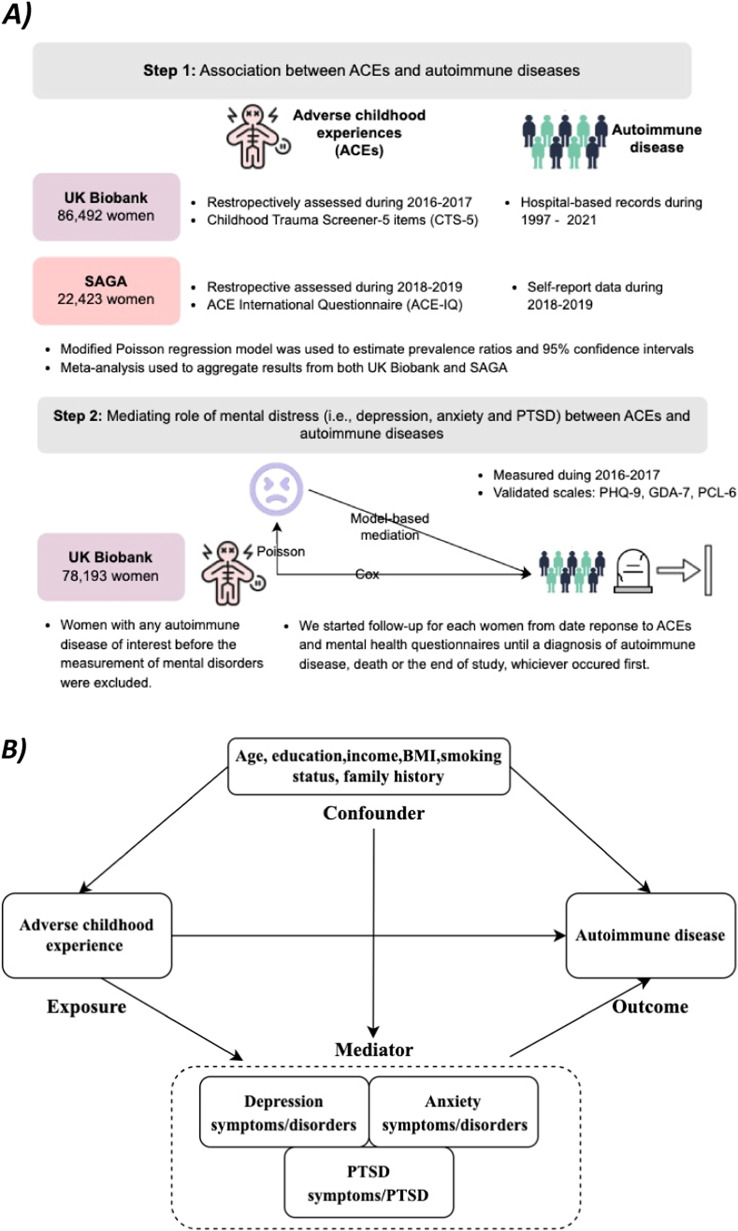
(A) Study design for the association analysis performed in the SAGA and UKB cohorts (step 1) and for the mediation analysis (step 2) performed in the UKB cohort and (B) a DAG presenting the relationships for the mediation analysis.

The SAGA cohort (https://afallasaga.is/english/) is a cohort study of women aged 18–69 years residing in Iceland in 2018 (Danielsdottir et al., 2022; Yang et al., 2022). All Icelandic speaking women were invited to participate in the study between March 2018 and July 2019. The participants of the SAGA cohort represented the general Icelandic female population in terms of age, education, geographical location, and monthly wages (Danielsdottir et al., 2022). We had cross-sectional data on women reporting on autoimmune diseases and, to align with data availability in the UKB, the five ACEs of interest (Figure 1).

### UK Biobank

The UKB represents a cohort of approximately 500,000 participants from the United Kingdom containing in-depth health information (Littlejohns et al., 2019). Participants aged 40–69 years were enrolled between 2006 and 2010 and followed since then. For the cross-sectional analyses and to be able to compare to the SAGA cohort, we included only women with complete information on ACEs (Figure 1). Cross-sectional baseline data (i.e., based on the individual inclusion date into the UKB in the period 2006–2010) were used for the analyses together with data from the SAGA cohort. Longitudinal data from the individual inclusion date until 2016–2017, when UKB participants filled out mental health questionnaires, were used for mediation analyses.

### Measurement of ACEs

ACEs were self-reported in both cohorts. In the SAGA cohort, ACEs were measured with a modified version of the ACE International Questionnaire (ACE-IQ) developed by the WHO assessing 13 ACEs before the age of 18 years: emotional neglect, physical neglect, emotional abuse, physical abuse, sexual abuse, domestic violence, living with a household member who abuses drugs and/or alcohol, living with a household member who is mentally ill or suicidal, incarceration of a household member, parental death or separation/divorce, being bullied, witnessing community violence, and exposure to war/collective violence (Organization, 2021).

In the UKB, 339,092 participants were invited to complete online mental health questionnaires during 2016 and 2017, including a retrospective measure of childhood maltreatment with the Childhood Trauma Screener-5 items (CTS-5). Of the invited participants, 46.4% (n = 157,366) responded to this online measure. The CTS-5 represents a 5-point Likert scale ranging from ‘never true’ to ‘very often true’ for the following types of ACEs: emotional neglect, physical neglect, emotional abuse, physical abuse, and sexual abuse. The threshold values to define each type of ACE are presented in Supplementary Tables 1A + B (Glaesmer et al., 2013). Hence, information from the CTS-5 completed in 2016–2017 was used for the primary analyses on the association between ACEs and autoimmune diseases.

To harmonize information on ACEs from both cohorts, we used the same 5 items of ACEs in the SAGA cohort. The primary exposure variable was the total number of ACEs (range 0 to 5) and each type of ACEs (0 [unexposed] versus 1 [exposed]). Rank order correlations between the different ACEs are presented for SAGA in Supplementary Figure 1 and for UKB in Supplementary Figure 2, which were calculated via Spearman coefficients.

### Autoimmune diseases

In the SAGA cohort, participants reported on whether they ever had been diagnosed with one or more of the following 12 autoimmune diseases (Supplementary Table 2): diabetes mellitus type 1, multiple sclerosis, Crohn’s disease, celiac disease, thyroid disease, Sjögrens disease, Addison’s disease, rheumatoid arthritis, systemic lupus erythematosus, polymyalgia rheumatica, psoriasis, and psoriatic arthritis. This occurred at the same time when participants reported on ACEs. A previous report using these data indicated null association between accumulation of 13 ACEs and multiple sclerosis (Gatto et al., 2022), yet we chose to include MS in the current analysis as one of the 12 autoimmune diseases as well as associations of the specific five ACEs assessed and MS in the current study.

In the UKB, autoimmune diseases were identified from hospital-based records using ICD-10 codes with these data available in the period from January 31, 1997, to January 31, 2021 (Supplementary Table 3).

### Mental health symptoms

In the UKB, we included information on symptoms of depression, anxiety, and PTSD. Among the 157,366 UKB participants, who had responded to online mental health questionnaires from 2016 to 2017, depressive symptoms were identified via the Patient-Health Questionnaire (PHQ-9) (Kroenke et al., 2001), anxiety symptoms via the Generalized Anxiety Disorder-7 scale (GAD-7) (Spitzer et al., 2006), and PTSD symptoms via the PTSD-Check-List-Civilian Short version (Wilkins et al., 2011) (PCL-S, Supplementary Table 4). These scales were filled out at the same time as the CTS-5 and we used both continuous scores and validated cut-offs for depression, anxiety, and PTSD, respectively. We removed all participants who did not answer at least 75% items of the questionnaires and the option ‘Prefer not to answer’ was treated as missing value. Single imputation was performed using predictive mean matching to replace missing values, utilizing the R packages ‘mice’.

### Covariates

We included the following covariates that were available in both cohorts: age (at responding, used as a continuous variable); educational level (categorized as without versus with college qualification); smoking status (previous, never, current); body mass index (BMI, normal weight [18.5–24.9] or abnormal weight [<18.5 or > 25]); and income (categorized into low income (SAGA: personal income every month ≤ISK 300,000; UKB: average total household income before tax < £18,000); medium income (SAGA: ISK 301,000–ISK 700,000; UKB: £18,000–£51,999); high income (SAGA: ≥ISK 700,001; UKB: ≥£52,000)).

### Statistical analyses

Descriptive numbers are presented as percentages or means with standard deviations (SD).

First, we used cross-sectional data (i.e., 2016–2017 in the UKB and 2018–2019 in the SAGA) and performed modified Poisson regression models with the sandwich variance estimators (Chen et al., 2018; Rogers & Stoner, 2015) to calculate the prevalence ratio (PR) and 95% confidence intervals (95%CIs) for the association between the total number of ACEs and any autoimmune disease. Similarly, we assessed the association between the number of ACEs, classified as 1 or ≥ 2 ACEs, and any autoimmune disease, always comparing to women without ACEs. We then estimated the associations between the total number of ACEs and 11 individual autoimmune diseases (Addison’s disease was excluded due to low case numbers). To determine associations between specific ACEs with any autoimmune disease, we first conducted separate analyses for each type of ACE, adjusting for all covariates and then additionally mutually adjusting for other types of ACEs (Yang et al., 2022). We conducted all the above analyses in both the SAGA and UKB. Finally, we meta-analyzed based on aggregated data from both SAGA and UKB with a random-effects model via the ‘metafor’ package (Viechtbauer, 2010).

Second, to study the potential mediation by mental distress in the association between ACEs and autoimmune diseases, we used longitudinal data from the UKB (Figure 1 presents a DAG for the model). We studied three potential mediators: self-reported depressive, anxiety, and PTSD symptoms. The date of filling out the online mental health questionnaires was used as the individual index date. We excluded women (n = 335) with more than 25% missing data on mental health symptoms (i.e., depression, anxiety, and PTSD) and women with any autoimmune diseases (n = 7,964) before the index date, leaving 78,193 women for the mediation analysis (Figure 1). Follow-up started at the date of filling out the mental health questionnaire during 2016–2017 and lasted until the occurrence of an autoimmune disease, death, or January 31, 2021, whichever came first. For the mediation analyses, we first assessed the associations between the total number of ACEs and categorized ACEs (i.e., 0,1, and ≥ 2), as well as any autoimmune disease using Cox regression models, represented as hazard ratios (HRs) including 95%CI. Secondly, we mutually adjusted for each mediator separately and for all mediators in the same model to observe changes in effect size. Next, we used a modified Poisson regression model to assess the associations between the total number of ACEs and ordinal ACEs (i.e., 0,1, and ≥ 2) with mediators. Furthermore, we employed a model-based mediation analysis framework using the “CMAverse’ package to estimate the percentage of mediators based on a weighted-based approach and bootstrap setting 100 times (Shi et al., 2021; VanderWeele & Vansteelandt, 2014). In addition, to further test the robustness of our results against the concern of reverse causality, we applied a 1-year lag time (i.e., started the follow-up 1 year after completing the mental health questionnaires). Finally, we conducted an additional mediation analysis by the history of registered clinical diagnosis of depression, anxiety, and stress-related disorders (retrieved based on ICD-10 code, F32, F33, F41, and F43) from 1997 until the onset of autoimmune diseases.

All analyses were adjusted for age, education, income, BMI, and smoking status and all statistical work was performed with R (version 4.1.1).

## Results

We included 22,423 women from the SAGA cohort (mean age 44 years) and 86,492 women from the UKB (mean age 55 years). Figure 1 and Supplementary Figure 3 present the study design and flow-chart, respectively. Characteristics for both cohorts are shown in Table 1, showing some important baseline differences in the educational level, income, BMI, and smoking status in addition to the age difference. At least one ACE was reported by 11,302 (50.4%) in the SAGA cohort and 30,038 (34.7%) in the UKB. Women who had experienced ACEs, compared to those without ACEs, were more likely to have a lower education and lower income, not be in a relationship, have a low or high BMI, and be smokers (Table 1).

**Table 1.** Characteristics of study population by the number of ACEs

	SAGA				UK Biobank			
	Overall	0 ACEs	1 ACE	≥2 ACEs	Overall	0 ACEs	1 ACE	≥2 ACEs
N	22,423	11,121	6,327	4,975	86,492	56,454	17,319	12,719
Age (Mean, SD)	43.81(13.51)	42.87(14.03)	45.07(13.20)	44.31(12.50)	55.42(7.65)	55.70(7.63)	55.29(7.63)	54.31(7.67)
Education level								
Without college qualification	9671(43.1)	4325(38.9)	2800(44.3)	2546(51.2)	41940(48.5)	27366(48.5)	8336(48.1)	6238(49.0)
With college qualification	12699(56.6)	6776(60.9)	3513(55.5)	2410(48.4)	38014(44.0)	24972(44.2)	7639(44.1)	5403(42.5)
Unknown	53(0.2)	20(0.2)	14(0.2)	19(0.4)	6538(7.6)	4116(7.3)	1344(7.8)	1078(8.5)
Income^a^								
Low	6359(28.4)	2957(26.6)	1762(27.8)	1640(33.0)	30406(35.2)	18936(33.5)	6335(36.6)	5135(40.4)
Medium	12081(53.9)	6040(54.3)	3452(54.6)	2589(52.0)	40129(46.4)	26586(47.1)	7957(45.9)	5586(43.9)
High	3215(14.3)	1704(15.3)	912(14.4)	599(12.0)	5478(6.3)	3797(6.7)	1030(5.9)	651(5.1)
Unknown	768(3.4)	420(3.8)	201(3.2)	147(3.0)	10479(12.1)	7135(12.6)	1997(11.5)	1347(10.6)
BMI^b^								
Normal	7967(35.5)	4312(38.8)	2106(33.3)	1549(31.1)	39068(45.2)	26337(46.7)	7713(44.5)	5018(39.5)
Abnormal	14203(63.3)	6680(60.1)	4143(65.5)	3380(67.9)	47220(54.6)	29986(53.1)	9562(55.2)	7672(60.3)
Unknown	253(1.1)	129(1.2)	78(1.2)	46(0.9)	204(0.2)	131(0.2)	44(0.3)	29(0.2)
Smoking status (%)								
Never	10946(48.8)	6578(59.1)	2828(44.7)	1540(31.0)	52919(61.2)	36429(64.5)	10039(58.0)	6451(50.7)
Previous	7972(35.6)	3237(29.1)	2463(38.9)	2272(45.7)	28052(32.4)	17070(30.2)	6050(34.9)	4932(38.8)
Current	3261(14.5)	1182(10.6)	969(15.3)	1110(22.3)	5315(6.1)	2839(5.0)	1190(6.9)	1286(10.1)
Unknown	244(1.1)	124(1.1)	67(1.1)	53(1.1)	206(0.2)	116(0.2)	40(0.2)	50(0.4)

Abbreviation: BMI, body mass index.

a Low income (SAGA: personal income every month ≤ISK 300,000; UK Biobank: average total household income before tax < £30,999); Medium income (SAGA: ISK 301,000-ISK 700,000; UK Biobank: £31,000–£100,000); High income (SAGA: ≥ISK 700,001; UK Biobank: ≥£100,000).

b Normal weight: BMI 18.5–24.9; Abnormal weight (Underweight: BMI < 18.5; Overweight: BMI 25.0–29.9; Obesity: BMI ≥ 30.0).

### Associations between ACEs and autoimmune diseases

An autoimmune disease was self-reported by 6,578 (29.3%) women in the SAGA while 11,217 (13.0%) had a hospital-based diagnosis in the UKB (Supplementary Figure 3). A higher number of ACEs was associated with increased prevalence of any autoimmune disease (SAGA: PR = 1.11 per ACE; 95%CI = 1.10–1.13; UKB: PR = 1.09 per ACE; 95%CI = 1.08–1.11). When pooling both cohorts, women with one ACE and ≥ 2 ACEs had a PR of 1.10 (95%CI = 1.06–1.13) and 1.29 (95%CI = 1.22–1.38), respectively (Table 2).

**Table 2.** Associations between self-reported ACEs and any autoimmune disease

			SAGA		UK Biobank		Overall
	Women,N	Case, N (%)	PR (95%CI)^a^	Women, N	Case, N (%)	PR (95%CI)^a^	PR (95%CI)^b^
The total number of ACEs	22,423	6,578 (29.3)	1.11(1.10–1.13)	86,492	11,217 (13.0)	1.09(1.08–1.11)	1.10 (1.08–1.12)
By number of ACEs							
0 ACEs	11,121	2,819 (25.3)	Ref	56,454	6,888 (12.2)	Ref	
1 ACE	6,327	1,909 (30.2)	1.11(1.05–1.16)	17,319	2,335 (13.5)	1.09(1.05–1.14)	1.10(1.06–1.13)
≥2 ACEs	4,975	1,850 (44.5)	1.34(1.28–1.41)	12,719	1,994 (15.7)	1.26(1.21–1.32)	1.29(1.22–1.38)

a Adjusted for age, education, income, BMI, smoking status;

b Aggregate results from both SAGA and UK

When studying the five specific ACEs, we found that all ACEs were associated with a higher PR of autoimmune diseases at a comparable level (PRs ranging between 1.18 and 1.27) (Supplementary Table 5). After additionally adjusting for other types of ACEs, sexual abuse, physical neglect, and emotional neglect remained significantly associated with any autoimmune disease (Supplementary Table 5).

**Figure 2.**
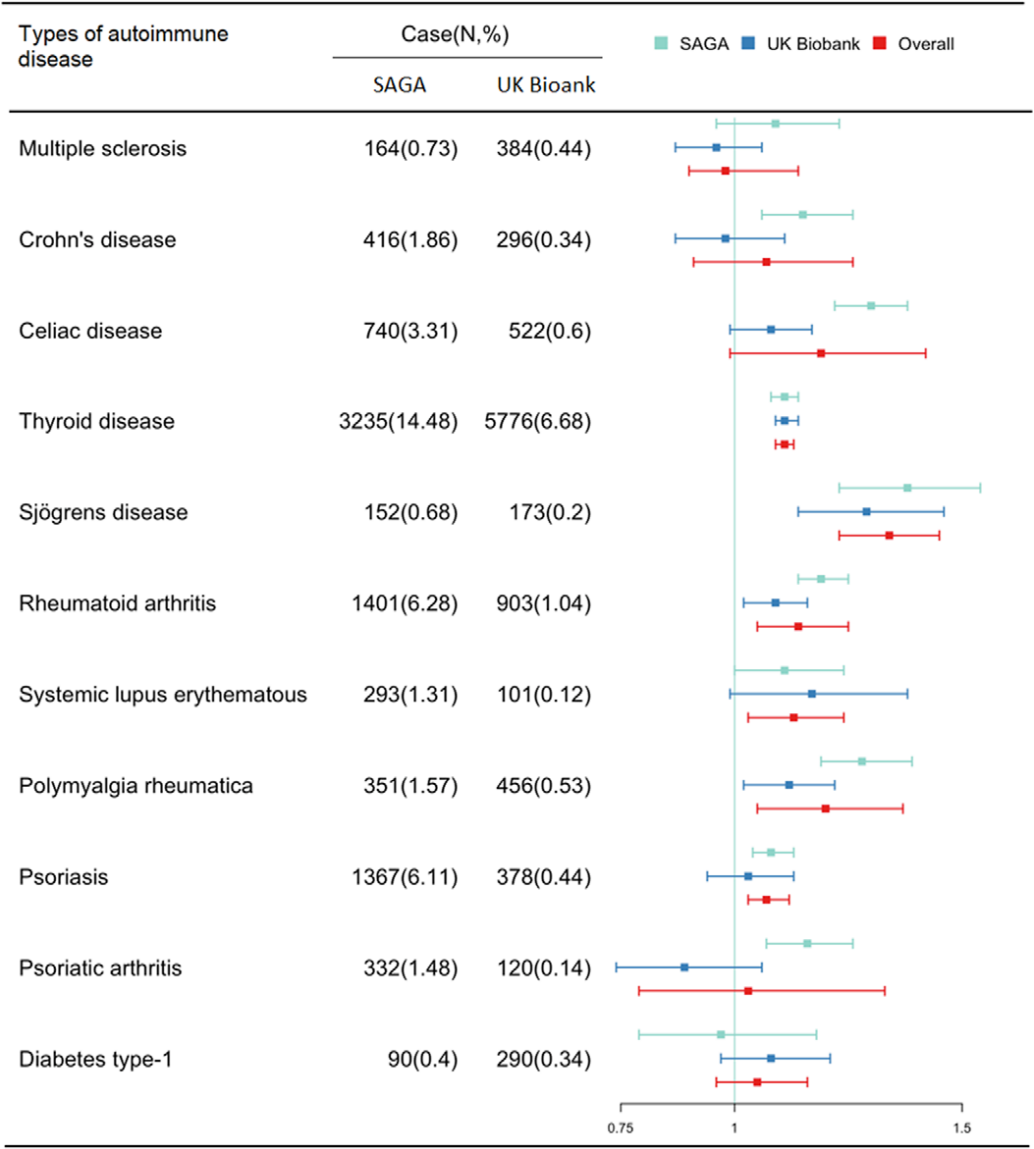
Associations between the total number of ACEs and specific autoimmune diseases. Abbreviation: BMI, body mass index; PR, prevalence ratio; 95%CI, 95% confidence interval. Adjusted for age, education, income, BMI, smoking status Addison”s disease is not included as a specific autoimmune disease due to the small sample size.

We found differences in the PRs for specific autoimmune diseases (Figure 2 and Supplementary Table 6). Women with ACEs, compared to those without, had a particular higher PR of thyroid disease, Sjögrens disease, rheumatoid arthritis, systemic lupus erythematous, polymyalgia rheumatica, and psoriasis, while we found no associations with other autoimmune diseases, such as multiple sclerosis, Crohn’s disease, or type-1 diabetes. Some of the results were slightly more pronounced in the SAGA cohort, particularly for celiac disease, rheumatoid arthritis, polymyalgia rheumatica, and psoriatic arthritis.

### Mediation analyses

During a mean follow-up of 4.2 (SD = 0.6) years among 78,193 UKB participants, we identified 3,204 new cases with any autoimmune disease. The number of cases was 2007, 672, and 525 among participants with 0,1, ≥2 ACEs, corresponding to a crude incidence rate of 9.20, 10.18, and 11.09 per 1000 person-years, respectively. As shown in Table 3, a higher number of ACEs were associated with an increased risk of any incident autoimmune disease (HR = 1.07, 95%CI 1.04–1.11). Compared with participants without ACEs, the HR of developing any autoimmune diseases was 1.10 (1.01–1.20) and 1.20 (1.09–1.33) for participants exposed to 1 or ≥ 2 ACEs, respectively. After additionally adjusting for each mediator and all mediators, the association attenuated slightly (Table 3). The majority of association between ACEs and any autoimmune diseases was mediated by depression, followed by PTSD and anxiety. In total, 28% of the association was mediated by the studied mental health symptoms. We obtained comparable results when a one-year lag time was used (Supplementary Table 7). A history of a clinical diagnosis of depression, anxiety, or stress-related disorder also mediated the associations between ACEs and autoimmune diseases yet to a much lesser extent (5%) than captured through corresponding symptoms (Supplementary Table 8).

**Table 3.** Estimated hazard ratios for the association between number of ACEs and autoimmune diseases

	Number of cases (incidence rate, per 1000 person years)	M0 model	M1: M0 + depression	M2: M0 + anxiety	M3: M0 + PTSD	M4: M0 + all
Total number of ACEs	3204/ (9.67)	1.07 (1.04–1.11)	1.05 (1.01–1.08)	1.06 (1.03–1.10)	1.05 (1.02–1.09)	1.04 (1.01–1.08)
Proportion mediated (%)		-	24.7% (19.4%–34.6%)	7.6% (6.9%–24.6%)	15.1% (4.3%–54.8%)	28.5% (10.7%–70.5%)
By number of ACEs						
0 ACEs	2007/ (9.20)	Ref	Ref	Ref	Ref	Ref
1 ACE	672/ (10.18)	1.10 (1.01–1.20)	1.08 (0.99–1.18)	1.09 (1.00–1.19)	1.09 (0.99–1.19)	1.07 (0.98–1.17)
≥2 ACEs	525/ (11.09)	1.20 (1.09–1.33)	1.13 (1.02–1.24)	1.17 (1.06–1.30)	1.15 (1.04–1.27)	1.11 (1.01–1.23)
Proportion mediated (%)		-	26.1% (16.1%–51.4%)	10.3% (5.1%–29.7%)	17.0% (6.3%–53.8%)	28.8% (13.5%–87.5%)

Abbreviation: BMI, body mass index; HR, hazard ratio; 95%CI, 95% confidence interval.

Depression symptoms were assessed with the 9-item Patient Health Questionnaire (PHQ-9) with a score of ≥10 as the cutoff for a probable case.

Anxiety symptoms were assessed with the 7-item Generalized Anxiety Disorders (GAD-7) with a score of ≥10 as the cutoff for a probable case.

PTSD symptoms were assessed with the 6-item Post-Traumatic Stress Disorder Checklist (PCL-6) with a score of ≥14 as the cutoff for a probable case.

M0: basic model, adjusted for age, education, income, BMI and smoking status.

M4: M0+ depression, anxiety and PTSD.

Proportion mediated: the proportion of the total effect that is mediated through the specific mediator.

## Discussion

The present study of two large cohorts including 108,915 women lends support to an association between ACEs and a higher prevalence of autoimmune diseases in adult women. Although the strength of the associations varied somewhat by the type of ACE and individual autoimmune disease, we observed a dose–response association between a higher number of ACEs and prevalence of autoimmune diseases in adulthood when adjusting for several important covariates. Furthermore, based on prospective data in the UK Biobank, we found that symptoms of depression, anxiety, and PTSD mediated approximately one-fourth of this association. Hence, the current findings indicate that ACEs are associated with modest risk of developing autoimmune diseases among women during adulthood and that mental distress might play an important mediating role. While most of the observed effect sizes were modest, these findings lend support to the role of traumatic exposure in early life in development of autoimmune disease requiring hospital admission decades later.

Our findings are of particular interest due to the comprehensive approach covering five severe and frequently studied ACEs (i.e., emotional neglect, physical neglect, emotional abuse, physical abuse, and sexual abuse) and a broad range of the most frequent autoimmune diseases. Furthermore, our findings are strengthened as both cohorts showed very similar results and we were able to meta-analyze the combined cohort. We found that particularly emotional neglect, physical neglect, and sexual abuse were associated with a higher prevalence of autoimmune diseases, but the results on physical abuse and emotional abuse also indicated a higher prevalence. Furthermore, we found that the elevation in prevalence by ACEs varied across the specific autoimmune diseases. Particularly increased prevalence rates by ACEs were observed for thyroid disease, Sjögrens disease, rheumatoid arthritis, systemic lupus erythematosus, psoriasis, and polymyalgia rheumatica while null associations were observed for multiple sclerosis, Crohn’s disease, psoriatic arthritis, and diabetes type-1. Previous studies have primarily investigated the correlation of any ACE with specific autoimmune diseases. Hence, the present study extends previous findings by studying five of the most severe ACEs with a broad range of autoimmune diseases. The results pattern indicating associations between ACEs with specific (but not all) autoimmune diseases requires confirmation in future studies; however, interestingly, the results pattern remained quite similar in both cohorts. Also, future research is needed to inform the potential varying weight of mental health morbidities in the associations between ACEs to specific autoimmune diseases.

Furthermore, the present study is the first to leverage detailed longitudinal data to test the potential mediating role of common mental health symptoms and disorders in the association between ACEs and autoimmune disease. The UKB provided longitudinal data to study this potential mediation in a large dataset with results suggesting that symptoms of depression, anxiety, and PTSD mediated approximately one fourth of the association between ACEs and autoimmune diseases. To our knowledge, only one recent study (Macarenco et al., 2021) found that the relationship between childhood trauma and autoimmune diseases was mediated by symptoms of dissociation and alexithymia. These findings lend further support to enhanced clinical alertness and treatment of mental distress among individuals with ACEs.

Potential mechanisms underlying the observed associations may cover a combination of psychological and biological mechanisms. The psychobiological stress of ACEs, for example, chronically increased inflammatory state (e.g., via a chronically increased hypothalamic–pituitary–adrenal (HPA) axis)(Heim et al., 2000), may persist into adulthood thereby affecting both adult mental health and the risk of developing an autoimmune disease. The detrimental influence of ACEs on mental health is well-described (Dragioti et al., 2022), which potentially affects the immune system and hence the risk of developing/mediating an autoimmune disease among individuals who have been exposed to ACEs. Furthermore, ACEs affect epigenetic DNA methylation which can persist into adulthood (Houtepen et al., 2018) and thereby affect the risk of developing an autoimmune disease and mental health distress.

### Strengths and limitations

The strengths of this study include the two large data-rich cohorts of women from the general population in Iceland and the UK yielding data on the association between five important ACEs and a total of 11 autoimmune diseases while controlling for a wide range of important covariates.

Regarding limitations, first, although pooling results from two large cohorts, several analyses were underpowered, such as on some specific autoimmune diseases. Second, ACEs were retrospectively assessed in adulthood (i.e., mean age of 44 and 55 years in the SAGA and UKB cohorts, respectively) and might therefore be subjective to recall bias. Studies have found retrospective information on ACEs to be rather reliable (Hardt et al., 2010; Yancura & Aldwin, 2009), although concurrent mental health symptoms may influence reporting (Colman et al., 2016). Yet, such bias is unlikely dependent on the outcome, that is, autoimmune disease, particularly when ascertained through health registers as confirmed clinical diagnosis. Third, autoimmune diseases were self-reported in the SAGA cohort but based on hospital-based records in the UKB. This might explain the different rates of autoimmune diseases as self-reporting might lead to over-reporting (e.g., reporting an autoimmune disease although not all specific diagnostic criteria have been met) while only using hospital-based codes may lead to under-estimation (e.g., the UKB might only detect the cases who were diagnosed in a hospital-based setting, although this should increase diagnostic accuracy). Yet, similar results obtained in both cohorts using these two different ascertainment strategies for autoimmune disease argue against substantial influence of this bias on the reported associations. In addition, as the UKB only had hospital-based data on autoimmune diseases, it is possible that some cases in the mediation analyses were not incident (e.g., previously diagnosed by their general practitioner). Fourth, the cohorts differed in terms of age and other characteristics, and we had no information on important variables, such as parental mental disorders or parental autoimmune diseases, which needs to be taken into account when interpreting the findings. Fifth, although the SAGA cohort has been reported to be representative of the general female population in Iceland, the UK Biobank population has reported to have higher socioeconomic status than the general population (Fry et al., 2017). Sixth, despite the comprehensive datasets, there is a risk of residual confounding due to unknown or unmeasured confounders including a risk for reverse causation.

## Conclusion

Within two large community-based cohorts covering a total of 108,915 women, we found associations between common ACEs and a higher prevalence of several specific autoimmune diseases among adult women. We found that these associations were partly mediated by adult self-reported symptoms of depression, anxiety, and PTSD. Hence, our findings lend support to the importance of ACEs in the etiology of autoimmune diseases and that mental health symptoms may play an important mediating role in these associations.

## Supporting information

Köhler-Forsberg et al. supplementary material Köhler-Forsberg et al. supplementary material

## Data Availability

The data used in this study are compiled in the Stress-And-Gene-Analysis (SAGA) cohort. According to the approval of the National Bioethics committee of Iceland, we cannot make the data publicly available. The SAGA cohort contains sensitive data and all use of data is restricted to scientific purposes only subjected to approval of the NBC (email: vsn@vsn.is). Interested researchers can obtain access to deidentified data by submitting a proposal to the SAGA cohort data management board (email: afallasaga@hi.is) which assists with submitting an amendment to the NBC. The UKB data can be applied for via direct contact to the UKB access team (email: access@ukbiobank.ac.uk).

## Supplementary material

The supplementary material for this article can be found at http://doi.org/10.1017/S0033291724003544.
