# Supplementary material for: Adverse childhood experiences, mental distress, and autoimmune disease in adult women: findings from two large cohort studies
Source: Psychol Med. 2025 Feb 11;55:e36. doi: 10.1017/S0033291724003544 (PMC12017369; doi:10.1017/S0033291724003544)
Supplement: Köhler-Forsberg et al. supplementary material [file S0033291724003544sup001.docx]

Table of Contents

[sTable1 (A) Participants’ response to the Childhood Trauma Screener-5 items (CTS-5) 2](#_Toc184648074)

[sTable1 (B) Items on the ACE-IQ used in this study 3](#_Toc184648075)

[sTable2 Details of autoimmune disease from SAGA cohort 4](#_Toc184648076)

[sTable3 The International Classification of Diseases (ICD) codes for diagnoses used in this study (UK Biobank) 5](#_Toc184648077)

[sTable4 Details of depression, anxiety and PTSD (UK Biobank) 7](#_Toc184648078)

[sTable5 Associations between different types of ACEs and any autoimmune disease 8](#_Toc184648079)

[sTable6 Associations between total number of ACEs and types of autoimmune disease 9](#_Toc184648080)

[sTable 7 Estimated hazard ratios for the association between number of ACEs and any autoimmune diseases, with the application of a 1-year lag time 10](#_Toc184648081)

[sTable 8: Estimated hazard ratios for the association between number of ACEs and any autoimmune diseases (history of mental disorders as mediators). 11](#_Toc184648082)

[sFigure1 Rank order correlations for type of ACEs (SAGA) 12](#_Toc184648083)

[sFigure2 Rank order correlations for type of ACEs (UK Biobank) 13](#_Toc184648084)

[sFigure3 The flow chart of study 14](#_Toc184648085)

sTable1 (A) Participants’ response to the Childhood Trauma Screener-5 items (CTS-5)

| Item | Never true | Rarely true | Sometimes  true | Often true | Very often  true |
| --- | --- | --- | --- | --- | --- |
| Physical abuse:  People in my family hit me so hard that it left me with bruises or marks | 124805(81.3) | 16467(10.7) | 10139(6.6) | 1329(0.9) | 861(0.6) |
| Emotional abuse:  I felt that someone in my family hated me | 129935(84.6) | 9315(6.1) | 10043(6.5) | 2357(1.5) | 1951(1.3) |
| Sexual abuse:  Someone molested me (sexually) | 140149(91.2) | 7113(4.6) | 4912(3.2) | 780(0.5) | 647(0.4) |
| Physical neglect:  There was someone to take me to the doctor if I needed it | 3288(2.1) | 1231(0.8) | 4118(2.7) | 16263(10.6) | 128701(83.8) |
| Emotional neglect:  I felt loved | 2164(1.4) | 7024(4.6) | 24829(16.2) | 39074(25.4) | 80510(52.4) |

Red fonts indicate the response that are categorized as ACEs

sTable1 (B) Items on the ACE-IQ used in this study

| Items | scoring |
| --- | --- |
| Physical abuse |  |
| Did a parent, guardian or other household member spank, slap, kick, punch or beat you up? | Never, Once, A few times, Many times |
| Did a parent, guardian or other household member hit or cut you with an object, such as a stick (or cane), bottle, club, knife, whip etc? | Never, Once, A few times, Many times |
| Emotional abuse |  |
| ﻿Did a parent, guardian or other household member yell, scream or swear at you, insult or humiliate you? | Never, Once, A few times, Many times |
| ﻿Did a parent, guardian or other household member threaten to, or actually, abandon you or throw you out of the house? | Never, Once, A few times, Many times |
| Sexual abuse |  |
| ﻿Did someone touch or fondle you in a sexual way when you did not want them to? | Never, Once, A few times, Many times |
| ﻿Did someone make you touch their body in a sexual way when you did not want them to? | ﻿ Never, Once, A few times, Many times |
| ﻿Did someone attempt oral, anal, or vaginal intercourse with you when you did not want them to? | Never, Once, A few times, Many times |
| ﻿Did someone actually have oral, anal, or vaginal intercourse with you when you did not want them to? | Never, Once, A few times, Many times |
| Physical neglect |  |
| ﻿How often did your parents/guardians not give you enough food  even when they could easily have done so? | Never, Once, A few times, Many times |
| ﻿Were your parents/guardians too drunk or intoxicated by drugs to take care of you? | Never, Once, A few times, Many times |
| ﻿How often did your parents/guardians not send you to school even when it was available? | Never, Once, A few times, Many times |
| Emotional neglect |  |
| ﻿Did your parents/guardians understand your problems and  worries? | ﻿Always, Most of the time, Sometimes, Rarely, Never |
| ﻿Did your parents/guardians really know what you were doing with  your free time when you were not at school or work? | Always, Most of the time, Sometimes, Rarely, Never |

Note: We used frequency version to calculate the ACE scores

sTable2 Details of autoimmune disease from SAGA cohort

| Items | Choices |
| --- | --- |
| Have you been diagnosed with diabetes? | 0=No, 1=Yes, treated with dietary changes, 2=Yes, treated with tablets, 3=Yes, treated with insulin incections, 4=Can/Will not answer |
| What type of diabetes do/did you have? | 0=Type 1 (most often originates during childhood), 1=Type 2 (most often diagnosed in adulthood), 2=Can /Will not answer |
| Have you been diagnosed with multiple sclerosis? | 0=No, 1=Yes, 2=Can/Will not answer |
| Have you been diagnosed with Crohn's disease? | 0=No, 1=Yes, 2=Can/Will not answer |
| Have you been diagnosed with Celiac disease? | 0=No, 1=Yes, 2=Can/Will not answer |
| Have you been diagnosed with thyroid disease? | 0=No, 1=Yes, 2=Can/Will not answer |
| Have you been diagnosed with Sjögrens disease? | 0=No, 1=Yes, 2=Can/Will not answer |
| Have you been diagnosed with Addisons disease? | 0=No, 1=Yes, 2=Can/Will not answer |
| Have you been diagnosed with rheumatoid arthritis? | 0=No, 1=Yes, 2=Can/Will not answer |
| Have you been diagnosed with systemic lupus erythematous? | 0=No, 1=Yes, 2=Can/Will not answer |
| Have you been diagnosed with polymyalgia rheumatica? | 0=No, 1=Yes, 2=Can/Will not answer |
| Have you been diagnosed with psoriasis? | 0=No, 1=Yes, 2=Can/Will not answer |
| Have you been diagnosed with psoriatic arthritis? | 0=No, 1=Yes, 2=Can/Will not answer |

sTable3 The International Classification of Diseases (ICD) codes for diagnoses used in this study (UK Biobank)

| Disease |  | ICD-10 |
| --- | --- | --- |
| Autoimmune disease | | |
| Disease of endocrine system | Diabetes mellitus, insulin dependent | E10 |
|  | Autoimmune thyroid disease | E03.5, E03.9, E05.0, E05.5, E05.9, E06.3, E06.5 |
|  | Addison’s disease | E27.1, E27.2 |
|  | Autoimmune polyglandular syndrome | E31.0 |
| Inflammatory arthritis | Reactive arthritis (Reiter’s syndrome) | M02.3, M02.8, M02.9, M05, M06, M08.0 |
|  | Rheumatoid arthritis | M08.1, M08.2, M08.3, M08.4 |
|  | Ankylosing spondylitis | M45 |
| Vasculitis | Polyarteritis nodosa and related condition (Incl. Kawasaki, Churg-Strauss syndrome, etc.) | M30 |
|  | Thrombotic microangiopathy | M31.1 |
|  | Granulomatosis with polyangiitis  (Wegeners’s granulomatosis) | M31.3 |
|  | Microscopic polyangiitis | M31.7 |
|  | Henoch-Schonlein purpura | D69.0 |
|  | Giant cell arteritis/Polymyalaia rheumatica | M35.3, M31.5, M31.6 |
| Connective tissue disorders | Systemic lupus erythematosus | M32 |
|  | Polymyositis/dermatomyositis | M33.0, M33.1, M33.2, M33.9 |
|  | Systematic sclerosis (scleroderma) | M34 |
|  | Sjogren’s syndrome | M35.0 |
|  | Mixed connective tissue disease | M35.1 |
|  | Behcet’s syndrome | M35.2 |
| Disease of skin system | Pemphigus anemia | L10.0 |
|  | Bullous pemphigoid | L12 |
|  | Dermatitis herpetiformis | L13.0 |
|  | Psoriasis | L40 |
|  | Alopecia areata | L64 |
|  | Vitiligo | L80 |
| Hematological disease | Pernicious anemia | D51.0 |
|  | Autoimmune hemolytic anemia | D59.0, D59.1 |
|  | Idiopathic thrombocytopenic purpura | D69.3 |
| Disease of nervous system | Acute disseminated encephalitis | G04 |
|  | Anti-NMDA receptor encephalitis | G13.1 |
|  | Multiple sclerosis | G35 |
|  | Neuromyelitis optica and ADEM | G36 |
|  | Guillain-Barre syndrome | G61.0, G61.1, G61.8, G61.9 |
|  | Myasthenia gravis | G70.0 |
| Disease of the digestive system | Primary biliary cirrhosis | K74.3 |
|  | Crohn’s disease | K50 |
|  | Ulcerative colitis | K51 |
|  | Celiac disease | K90.0 |
| Others | Acute rheumatic fever and chorea | I00, I01.0, I01.1, I01.2, I01.8, I0I.9, I02.0, I02.9 |
|  | Sarcoidosis | D86 |
|  | IgA nephropathy | N00, N01, N03, N05 |

sTable4 Details of depression, anxiety and PTSD (UK Biobank)

| Questions | Field code |
| --- | --- |
| Items of depression (PHQ-9) | |
| Little interest or pleasure in doing things | 20514 |
| Feeling down, depressed, or hopeless | 20510 |
| Trouble sleeping | 20517 |
| Feeling tired | 20519 |
| Poor appetite or overeating | 20511 |
| Feeling bad about yourself | 20507 |
| Trouble concentrating | 20508 |
| Moving or speaking slowly or fidgety or restless | 20518 |
| Thoughts that you would be better off dead | 20513 |
| Items of anxiety (GAD-7) | |
| Feeling nervous, anxious or on edge | 20506 |
| Not being able to stop or control worrying | 20509 |
| Worrying too much about different things | 20520 |
| Trouble relaxing | 20515 |
| Being so restless that it is hard to sit still | 20516 |
| Becoming easily annoyed or irritable | 20505 |
| Feeling afraid as if something awful might happen | 20512 |
| Items of PTSD (PCL-S) | |
| Repeated disturbing thoughts of stressful experience in past month | 20497 |
| Felt very upset when reminded of stressful experience in past month | 20498 |
| Avoided activities or situations because of previous stressful experience in past month | 20495 |
| Felt distant from other people in past month | 20496 |
| Felt irritable or had angry outbursts in past month | 20494 |
| Trouble concentrating | 20508 |

sTable5 Associations between different types of ACEs and any autoimmune disease

|  | SAGA |  | UK Biobank |  | Overall | |
| --- | --- | --- | --- | --- | --- | --- |
|  | PR (95%CI)^a^ | PR (95%CI)^b^ | PR (95%CI) ^a^ | PR (95%CI) ^b^ | PR (95%CI)^a^ | PR (95%CI)^b^ |
| Physical abuse |  |  |  |  |  |  |
| No | Ref | Ref | Ref | Ref | Ref | Ref |
| Yes | 1.25(1.17-1.35) | 1.02(0.94-1.10) | 1.24(1.18-1.32) | 1.12(1.05-1.20) | 1.25(1.19-1.30) | 1.07(0.98-1.18) |
| Emotional abuse |  |  |  |  |  |  |
| No | Ref | Ref | Ref | Ref | Ref | Ref |
| Yes | 1.28(1.21-1.34) | 1.15(1.08-1.22) | 1.17(1.11-1.23) | 1.03(0.97-1.09) | 1.22(1.13-1.33) | 1.09(0.98-1.21) |
| Sexual abuse |  |  |  |  |  |  |
| No | Ref | Ref | Ref | Ref | Ref | Ref |
| Yes | 1.20(1.14-1.25) | 1.14(1.09-1.19) | 1.16(1.1-1.22) | 1.09(1.04-1.15) | 1.18(1.14-1.22) | 1.12(1.07-1.17) |
| Physical neglect |  |  |  |  |  |  |
| No | Ref | Ref | Ref | Ref | Ref | Ref |
| Yes | 1.25(1.16-1.33) | 1.10(1.02-1.18) | 1.29(1.21-1.37) | 1.18(1.11-1.26) | 1.27(1.21-1.33) | 1.14(1.06-1.23) |
| Emotional neglect |  |  |  |  |  |  |
| No | Ref | Ref | Ref | Ref | Ref | Ref |
| Yes | 1.21(1.15-1.26) | 1.11(1.06-1.17) | 1.16(1.12-1.21) | 1.08(1.03-1.13) | 1.18(1.14-1.22) | 1.09(1.06-1.13) |

a. Adjusted for age, education, income, BMI and smoking status.

b. additionally adjusted for other types of ACEs;

sTable6 Associations between total number of ACEs and types of autoimmune disease

| Types of autoimmune disease | SAGA | UK Biobank | Overall |
| --- | --- | --- | --- |
|  | **PR (95%CI)** | **PR (95%CI)** | **PR (95%CI)** |
| Multiple sclerosis | 1.09(0.96-1.23) | 0.96(0.87-1.06) | 0.98(0.90-1.14) |
| Crohn's disease | 1.15(1.06-1.26) | 0.98(0.87-1.11) | 1.07(0.91-1.26) |
| Celiac disease | 1.30(1.22-1.38) | 1.08(0.99-1.17) | 1.19(0.99-1.42) |
| Thyroid disease | 1.11(1.08-1.14) | 1.11(1.09-1.14) | 1.11(1.09-1.13) |
| Sjögrens disease | 1.38(1.23-1.54) | 1.29(1.14-1.46) | 1.34(1.23-1.45) |
| Rheumatoid arthritis | 1.19(1.14-1.25) | 1.09(1.02-1.16) | 1.14(1.05-1.25) |
| Systemic lupus erythematous | 1.11(1.00-1.24) | 1.17(0.99-1.38) | 1.13(1.03-1.24) |
| Polymyalgia rheumatica | 1.28(1.19-1.39) | 1.12(1.02-1.22) | 1.20(1.05-1.37) |
| Psoriasis | 1.08(1.04-1.13) | 1.03(0.94-1.13) | 1.07(1.03-1.12) |
| Psoriatic arthritis | 1.16(1.07-1.26) | 0.89(0.74-1.06) | 1.03(0.79-1.33) |
| Diabetes type-1 | 0.97(0.79-1.18) | 1.08(0.97-1.21) | 1.05(0.96-1.16) |

Adjusted for age, education, income, BMI and smoking status.

sTable 7 Estimated hazard ratios for the association between number of ACEs and any autoimmune diseases, with the application of a 1-year lag time

|  | **Number of cases**  **(incidence rate, per 1000 person years)** | **M0 model**  **HR (95% CI)** | **M1:**  **M0+depression**  **HR (95% CI)** | **M2:**  **M0+anxiety**  **HR (95% CI)** | **M3:**  **M0+PTSD**  **HR (95% CI)** | **M4:**  **M0+all**  **HR (95% CI)** |
| --- | --- | --- | --- | --- | --- | --- |
| **Total number of ACEs** | 2487/ (7.51) | 1.07 (1.03-1.12) | 1.05 (1.01-1.09) | 1.07 (1.03-1.11) | 1.05 (1.01-1.10) | 1.05 (1.01-1.09) |
| **Proportion mediated (%)** |  | - | 18.5% (11.9%-29.6%) | 7.6% (1.8%-12.7%) | 16.0% (4.7%-23.4%) | 25.1% (14.7%-47.9%) |
|  |  |  |  |  |  |  |
| **By number of ACEs** |  |  |  |  |  |  |
| 0 ACEs | 1566/ (7.18) | Ref | Ref | Ref | Ref | Ref |
| 1 ACE | 511/ (7.75) | 1.08 (0.97-1.19) | 1.06 (0.96-1.17) | 1.07 (0.97-1.18) | 1.06 (0.96-1.17) | 1.05 (0.95-1.16) |
| ≥2 ACEs | 410/ (8.67) | 1.21 (1.09-1.36) | 1.15 (1.03-1.29) | 1.19 (1.06-1.33) | 1.15 (1.03-1.29) | 1.13 (1.01-1.26) |
| **Proportion mediated (%)** |  | - | 26.5% (11.6%-92.7%) | 11.1% (2.0%-39.8%) | 22.8% (8.4%-80.5%) | 23.0% (17.1%-67.4%) |

Abbreviation: BMI, body mass index; HR, hazard ratio; 95%CI, 95% confidence interval

Depression symptoms were assessed with the 9-item Patient Health Questionnaire (PHQ-9) with a score of ≥10 as the cutoff for a probable case.

Anxiety symptoms were assessed with the 7-item Generalized Anxiety Disorders (GAD-7) with a score of ≥10 as the cutoff for a probable case.

PTSD symptoms were assessed with the 6-item Post-Traumatic Stress Disorder Checklist (PCL-6) with a score of ≥14 as the cutoff for a probable case.

M0: basic model, adjusted for age, education, income, BMI and smoking status.

M4: M0+ depression, anxiety and PTSD.

Proportion mediated: the proportion of the total effect that is mediated through the specific mediator.

sTable 8: Estimated hazard ratios for the association between number of ACEs and any autoimmune diseases (history of mental disorders as mediators).

|  | Number of cases  (incidence rate, per 1000 person years) | Basic model  HR (95%CI) | Additionally adjusted for history of mental disorders  HR (95%CI) |
| --- | --- | --- | --- |
| Total number of ACEs | 3204/ (9.67) | 1.07 (1.04-1.11) | 1.07 (1.03-1.10) |
| Proportion mediated (%) |  | - | 5.0% (2.7%-6.3%) |
|  |  |  |  |
| By number of ACEs |  |  |  |
| 0 ACEs | 2007/ (9.20) | Ref | Ref |
| 1 ACE | 672/ (10.18) | 1.10 (1.01-1.20) | 1.10 (1.01-1.20) |
| ≥2 ACEs | 525/ (11.09) | 1.20 (1.09-1.33) | 1.19 (1.08-1.31) |
| Proportion mediated (%) |  | - | 5.0% (2.8%-6.0%) |

*basic model, adjusted for age, education, income, BMI and smoking status.*

*Proportion mediated: the proportion of the total effect that is mediated through the specific mediator*

*2,497 participants with a history of depression, anxiety and stress-related disorder*


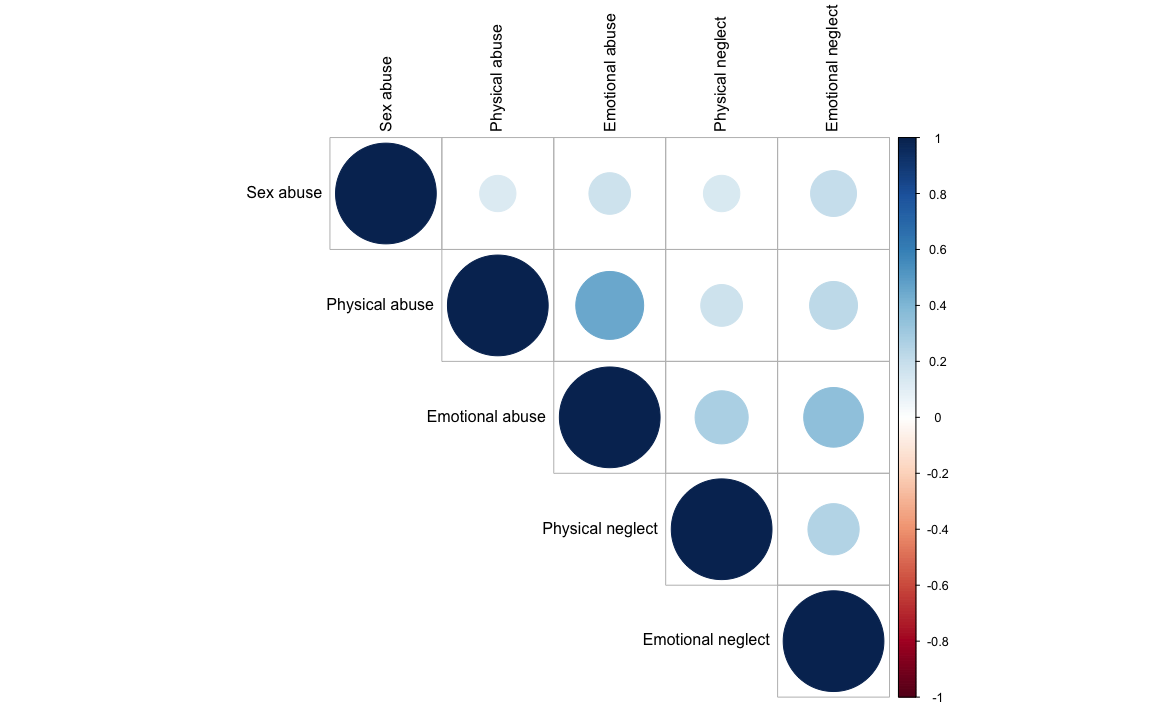


sFigure1 Rank order correlations for type of ACEs (SAGA)


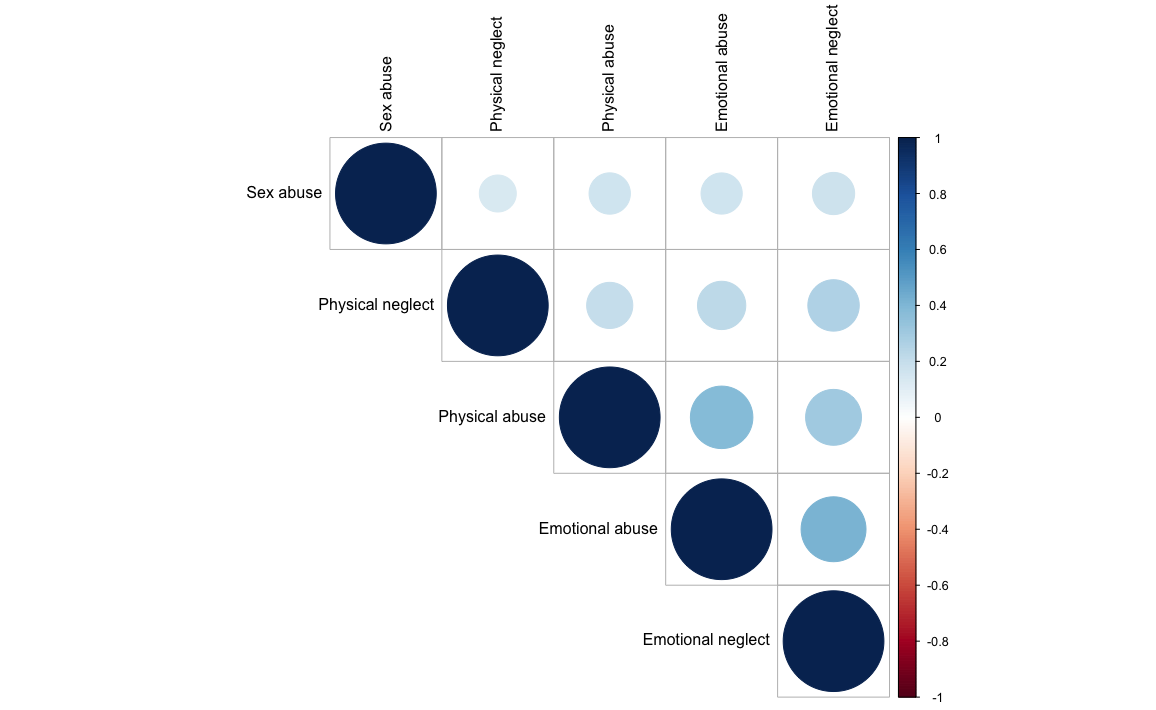


# sFigure2 Rank order correlations for type of ACEs (UK Biobank)


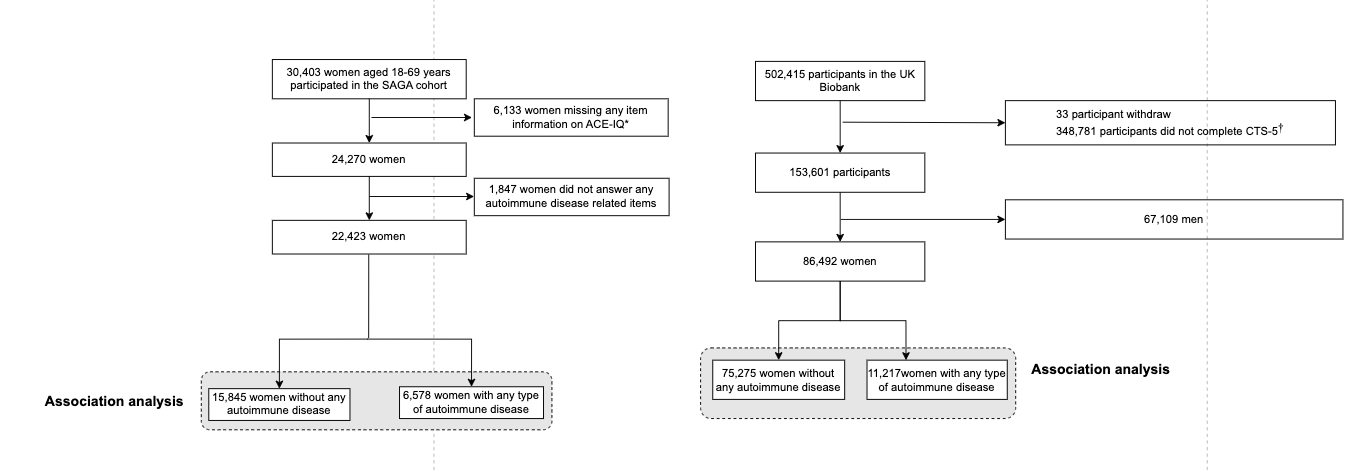


sFigure3 The flow chart of study
